# Autoradiographic Mapping of 5-**H** **T**_1**B**/1**D**_ Binding Sites in the Rhesus Monkey Brain Using [*carbonyl*-^11^C]zolmitriptan

**DOI:** 10.1155/2011/694179

**Published:** 2011-10-12

**Authors:** Örjan Lindhe, Per Almqvist, Matts Kågedal, Sven-Åke Gustafsson, Mats Bergström, Dag Nilsson, Gunnar Antoni

**Affiliations:** ^1^Uppsala Imanet AB, GE Healthcare, P.O. Box 967, 751 09 Uppsala, Sweden; ^2^Department of Clinical Neuroscience, Karolinska Institute, 171 77 Stockholm, Sweden; ^3^AstraZeneca R&D, 151 85 Södertälje, Sweden; ^4^Preclinical PET Platform, Department of Medicinal Chemistry, Uppsala University, Box 574, 75123 Uppsala, Sweden

## Abstract

Zolmitriptan is a serotonin 5-HT_1B/1D_ receptor agonist that is an effective and well-tolerated drug for migraine treatment. In a human positron emission tomography study, [^11^C]zolmitriptan crossed the blood-brain barrier but no clear pattern of regional uptake was discernable. The objective of this study was to map the binding of [^11^C]zolmitriptan in Rhesus monkey brain using whole hemisphere *in vitro* autoradiography with [^11^C]zolmitriptan as a radioligand. In saturation studies, [^11^C]zolmitriptan showed specific (90%) binding to a population of high-affinity binding sites (Kd 0.95–5.06 nM). There was regional distribution of binding sites with the highest density in the ventral pallidum, followed by the external globus pallidus, substantia nigra, visual cortex, and nucleus accumbens. In competitive binding studies with 5-HT_1_ receptor antagonists, [^11^C]zolmitriptan binding was blocked by selective 5-HT_1B_ and 5-HT_1D_ ligands in all target areas. There was no appreciable change in binding with the addition of a 5-HT_1A_ receptor antagonist.

## 1. Introduction

Triptans act as 5-hydroxytryptamine, 5-HT_1B_ and 5-HT_1D_, receptors agonists, and in some cases also activate 5-HT_1F_ receptors [1]. Zolmitriptan is a 5-HT_1B/1D_ receptor agonist, which has actions at the peripheral [2] and central ends of the trigeminovascular system [3–5]. Zolmitriptan is an effective and well-tolerated acute treatment of migraine [6], with oral and intranasal formulations [7]. Triptans are believed to exert an antimigraine action by activating 5-HT_1_ receptors in vascular structures of the brain and meninges [8, 9]. Besides these vascular actions, zolmitriptan has been suggested to act at the trigeminal nucleus and higher pain centers of the brain [4, 5, 10, 11].

In a previous study using [*carbonyl*-^11^C]zolmitriptan ([^11^C]zolmitriptan), positron emission tomography (PET) imaging was used to describe a rapid uptake of [^11^C]zolmitriptan into the brain through the blood-brain barrier (BBB) [12]. Zolmitriptan was found in all brain areas studied at concentrations compatible with pharmacological activity. However, no clear difference in the regional uptake of [^11^C]zolmitriptan, correlating with the known distribution of 5-HT_1B/1D_ receptors, was seen.

Autoradiography on whole-hemisphere brain cryosections provides images with high resolution and is therefore a suitable technique for the detailed description of receptor binding sites [13]. Such autoradiographic images can also serve as high resolution anatomical correlates for lower resolution PET and single photon emission computed tomography receptor studies. In the cat brain, [^3^H]sumatriptan [14] and [^3^H]zolmitriptan [5] bound to the nucleus tractus solitarius, to the trigeminal nucleus caudalis in the brainstem, and in the dorsal horns of the C1 and C2 cervical spinal cord. Competition studies excluded binding to 5-HT_1A_ and 5-HT_1F_ and confirmed binding to 5-HT_1B_ and 5-HT_1D_ receptors, although the relative contribution of these two receptor subtypes to the total binding of [^3^H]zolmitriptan was not elucidated. In the postmortem human brain stem and spinal cord, [^3^H]sumatriptan showed a distribution of high-density binding similar to that in the cat. The substantia nigra and layer V of the frontal cortex showed high specific binding, followed by the globus pallidus interna and externa [15].

The aim of the present study was to map the binding sites of [^11^C]zolmitriptan in the Rhesus monkey brain, and to characterize the regional distribution of zolmitriptan-binding 5-HT_1_ receptor subtypes. It has been suggested that 5-HT_1B_ receptors predominate over 5-HT_1D_ receptors in the human brain [16]. Selective ligands are now available to distinguish between 5-HT_1_ receptor subtypes. Here we have used WAY-100635, SB224289, and BRL15572 to explore differential binding of [^11^C]zolmitriptan to 5-HT_1A_, 5-HT_1B_ and 5-HT_1D_ receptors, respectively.

## 2. Methods

### 2.1. Chemicals

#### 2.1.1. Zolmitriptan

**Figure 1:**
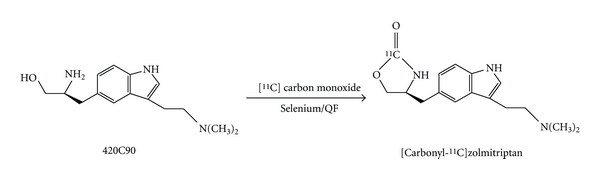
Preparation of [*carbonyl*-^11^C]zolmitriptan.

Zolmitriptan [(S)-4-[[3-[2-(dimethylamino)ethyl]-1Hindol-5-yl]methyl]-2-oxazolidinone] was provided by AstraZeneca (Alderly Park, UK). [*Carbonyl*-^11^C]zolmitriptan ([^11^C]zolmitriptan) was synthesized according to a standard manufacturing procedure developed at Uppsala University. [^11^C]carbon dioxide was produced by irradiation of nitrogen gas by 17 MeV protons from a Scanditronix MC-17 cyclotron. The [^11^C]carbon dioxide was converted to [^11^C]carbon monoxide and used in a selenium mediated carbonylation reaction in an autoclave [17] according to Figure 1. After chromatographic purification the product was dissolved in saline (NaCl 9 mg/mL)/99.5% EtOH, 90/10 v/v.

#### 2.1.2. WAY 100635

WAY 100635 [N-(2-(4-(2-methoxyphenyl)-1-piperazinyl)ethyl)-N-(2-pyridyl)-cyclohexanecarboxamide trichloride] (Sigma-Aldrich, St Louis, USA). [*carbonyl-* ^11^C]WAY 100635 ([^11^C]WAY) was synthesized as previously described [18].

#### 2.1.3. SB224289

SB224289 [1′-Methyl-5-[[2′-methyl-4′-(5-methyl-1,2,4-oxadiazol-3-yl)biphenyl-4-yl]carbonyl]-2,3,6,7-tetrahydrospiro[furo[2,3-f]indole-3,4′-piperidine hydrochloride], Tocris Cookson Ltd (Bio Nuclear AB, Bromma, Sweden). SB224289 is a selective 5-HT_1B_ receptor antagonist (*pK*_*i*_ = 8.2) that displays greater than 60-fold selectivity over 5-HT_1D_, 5-HT_1A_, 5-HT_1E_, 5-HT_1F_, 5-HT_2A_ and 5-HT_2C_ receptors in radioligand binding and functional assays (according to the supplier). SB224289 is centrally active following oral administration *in vivo*.

#### 2.1.4. BRL15572

BRL15572 [(3-[4-(4-Chlorophenyl)piperazin-1-yl]-1,1-diphenyl-2-propanol hydrochloride], Tocris Cookson Ltd (Bio Nuclear AB, Bromma, Sweden). BRL15572 is a selective h5-HT_1D_ antagonist that displays 60-fold selectivity over 5-HT_1B_ and exhibits little or no affinity for a range of other receptor types (according to the supplier).

### 2.2. Tissue

Brain tissue specimens from Rhesus monkeys (*Macaca mulatta*) were stored frozen at −70°C. The tissue specimen comprised whole brain hemispheres (right and left), including the cerebrum, cerebellum, and mesencephalon. Coronal cryostat sections (30 *μ*m) from the brain hemispheres were mounted on glass slides and air-dried at room temperature. A total of 120 tissue sections (each section separated by 0.5 mm) were used for the detection of zolmitriptan binding. The slides were stored at −20°C until use.

### 2.3. Autoradiography

Tissue sections were first pre-incubated in a Tris-buffer (50 mM Tris-HCl buffer pH 7.4, 120 mM NaCl, 5 mM KCl) for 10 minutes at room temperature. The incubations were performed at room temperature in the Tris-buffer with 2 mM CaCl_2_, 1 mM MgCl_2_, 0.01% ascorbate, 10 *μ*M pargyline, and with [^11^C]zolmitriptan. Concentrations of [^11^C]zolmitriptan and [^11^C]WAY were 2.5–3 nM for studies of regional distribution and 0.1–30 nM of [^11^C]zolmitriptan for estimations of Bmax and Kd.

For competition of radioligand binding the following compounds were used: zolmitriptan, serotonin, WAY100635, SB224289, and BRL15572. Incubations with the blocking agents were done at concentrations of 0.01, 0.1, 1, and 10 *μ*M. After radiotracer incubation, tissue slides were rinsed in Tris-buffer (3 × 3 minutes), carefully dried at 37°C, and placed on phosphor image plates (Molecular Dynamics, USA) together with reference samples (20 *μ*L aliquots taken from the incubation buffer) for a minimum of 40 minutes exposure, and scanned in a Phosphor Imager Model 400S (Molecular Dynamics, USA).

### 2.4. Anatomical Localization of Binding Sites and Quantification of [^11^C]zolmitriptan Binding

The autoradiographic images were digitized and superimposed on pictures of the corresponding tissue section by using image analysis (Adobe PhotoShop) in order to correlate the areas of tracer binding to anatomical structures. Evaluation of the Rhesus monkey brain anatomy was done according to Paxinos et al. [19]. The specific [^11^C]zolmitriptan binding was calculated as the difference between total and nonspecific binding and expressed in pmol/g wet tissue.

## 3. Results

### 3.1. Pharmacology

In all brain areas, [^11^C]zolmitriptan binding was completely inhibited by the addition of high concentration (10 *μ*M) serotonin at 1 nM tracer concentration and was ≥90% inhibited at 3 nM tracer. At this tracer concentration, 0.1 *μ*M zolmitriptan blocked ≥90% tracer binding (data not shown). The correlation between incubation time and specific tracer binding was analyzed and found to reach a plateau value at 30 minutes using 30 nM of [^11^C]zolmitriptan. Extending the incubation time further did not result in increased specific [^11^C]zolmitriptan binding. By using serotonin and unlabeled zolmitriptan to inhibit binding of [^11^C]zolmitriptan to brain slices, its targets were proven to be serotonin receptors with specificity for zolmitriptan. Less than 10% of the binding was nonspecific (data not shown).

A semi-quantitative saturation analysis indicated the lowest Kd in the substantia nigra (1.0 nM), followed by the visual cortex (1.1 nM), the external globus pallidus (1.3 nM), the nucleus accumbens (1.8 nM), the ventral pallidum (2.2 nM), the cerebellum (4.9 nM), and the frontal cortex (5.1 nM) (Table 1).

### 3.2. Distribution of [^11^C]Zolmitriptan Binding

**Figure 2:**
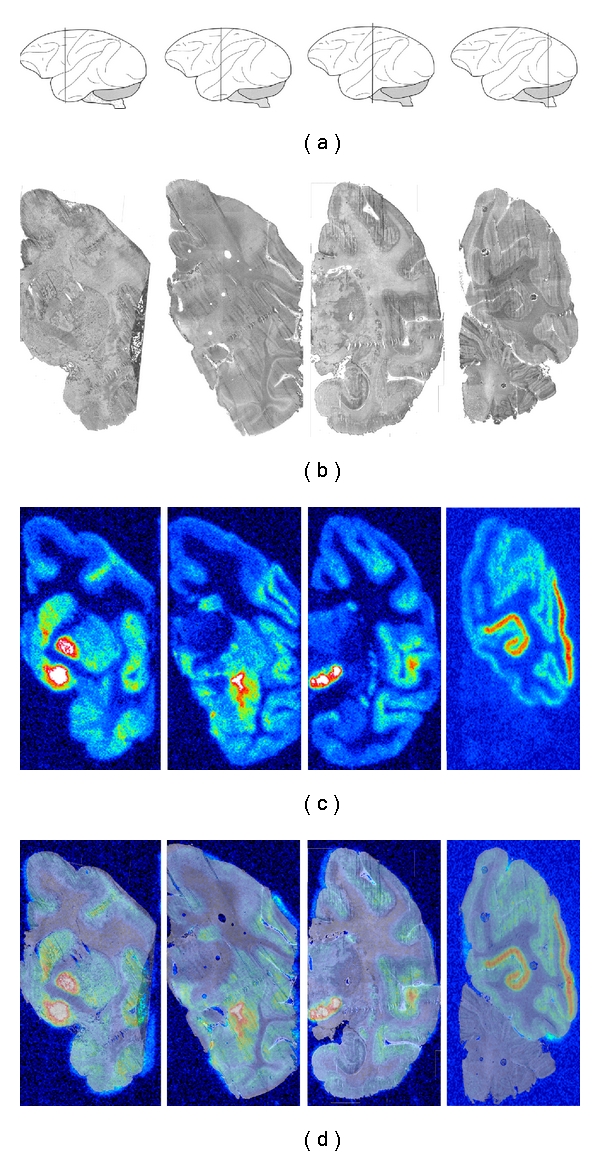
Color-coded autoradiograms showing Rhesus monkey whole-hemisphere autoradiography using [^11^C]zolmitriptan. (a) Schematic drawings of the Rhesus monkey brain according to Paxinos et al. [19] to show the anterior-posterior position of the selected coronal tissue sections; (b) corresponding tissue sections; (c) autoradiograms; (d) superimposed autoradiograms and corresponding tissue sections for better anatomical localization of [^11^C]zolmitriptan binding.

**Figure 3:**
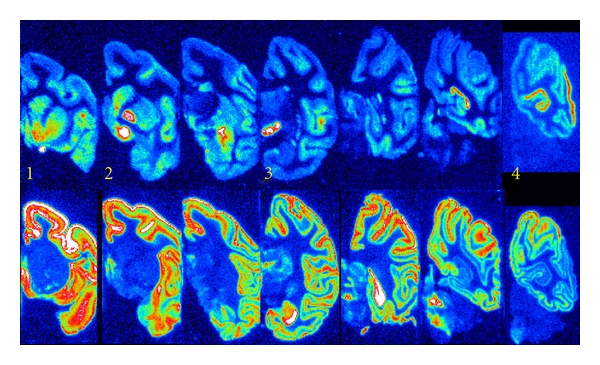
Color-coded autoradiograms of [^11^C]zolmitriptan (top) and [^11^C]WAY 100635 (bottom) on consecutive brain sections, comparing the distribution of binding sites for the respective tracer. Numbers identify sections where strong [^11^C]zolmitriptan binding is observed. (1) Nucleus accumbens, (2) Ventral pallidum and external globus pallidus (3) Substantia nigra, and (4) Visual cortex. Note the minimal overlap of tracer binding sites.

A regional distribution of [^11^C]zolmitriptan binding was detected in the Rhesus monkey brain tissue sections, with several high-density binding sites within well-defined anatomical areas (Figure 2). The highest densities of tracer binding were found in (i) the ventral pallidum (17.9 ± 2.6 pmol/g wet tissue), i.e. those parts of the globus pallidus located inferior to the anterior commissure, (ii) in the globus pallidus externa (12.2 ± 1.4 pmol/g wet tissue) and interna, and (iii) in the substantia nigra pars compacta and pars reticulata (11.4 ± 1.6 pmol/g wet tissue) (Figure 2). In addition, high-density binding was located in the visual cortex (9.8 ± 1.5 pmol/g wet tissue), including the calcarine fissure and the lateral cortex of the occipital pole (Figure 2) and in the nucleus accumbens (9.5 ± 0.6 pmol/g wet tissue), that is, where the head of the caudate and the anterior portion of the putamen meet just lateral to the septum pellucidum (Figure 3). [^11^C]Zolmitriptan binding was low in the frontal cortex (5.2 ± 0.8 pmol/g wet tissue) and virtually absent in the cerebellum (1.6 ± 0.4 pmol/g wet tissue). The distribution and the density of tracer binding were highly consistent in the 8 whole-hemisphere tissue samples used for this study. Neither the pons nor the medulla oblongata was included in the brain tissue specimen used for this study and potential tracer binding to these parts of the brain stem was not explored.

### 3.3. Comparing Distribution of [^11^C]Zolmitriptan and [^11^C]WAY-100635 Binding Sites

High-density binding of [^11^C]WAY-100635 was present in most neocortical areas of the Rhesus monkey brain, with lower levels in the occipital cortex. The basal ganglia (nucleus caudatus, putamen, pallidum) and thalamus were virtually devoid of 5-HT_1A_ receptor binding (Figure 2). When comparing the distribution of binding sites for [^11^C]zolmitriptan and [^11^C]WAY-100635 on consecutive tissue sections, there was very limited overlap of binding sites. Anatomical areas with high-density binding of [^11^C]WAY-100635 had no or very low levels of [^11^C]zolmitriptan binding and vice versa. While there was some overlap of tracer binding in the neocortex, the visual cortex of the occipital lobe showed exclusive [^11^C]zolmitriptan binding. Very low binding was obtained in the cerebellum with either tracer, indicating that the cerebellar cortex was virtually devoid of these binding sites (Figure 2). Competitive binding with WAY 100635 reduced [^11^C]zolmitriptan binding in the substantia nigra and the visual cortex by approximately 20% at 1.0 *μ*M concentration, while tracer binding to other brain areas tested was unaffected (Table 1).

### 3.4. Distribution of 5-HT_1B_ and 5-HT_1D_ Receptors in the Rhesus Monkey Brain

Differential receptor binding of [^11^C]zolmitriptan was explored (Table 2). The selective 5-HT_1B_ receptor antagonist SB224289 blocked 50–80% of [^11^C]zolmitriptan binding at a 1 *μ*M concentration. [^11^C]zolmitriptan binding was reduced by 70–80% in the substantia nigra, the external globus pallidus and the visual cortex, and by approximately 50% in the nucleus accumbens and the frontal cortex. In contrast, selective 5-HT_1D_ antagonist BRL15572 had much less of an effect in all areas tested, blocking ≤25% of [^11^C]zolmitriptan binding at a 1 *μ*M concentration (Table 2).

## 4. Discussion

The distribution of [^11^C]zolmitriptan binding sites in the Rhesus monkey brain was consistent with previously reported distribution of 5-HT_1B_ and 5-HT_1D_ receptors in the postmortem human brain, using whole-hemisphere autoradiography and the radioligand [^3^H]GR 125743 [16]. Similar to [^3^H]GR 125743, [^11^C]zolmitriptan binding was highest in the substantia nigra and the globus pallidus. Lower levels were detected in the striatum, with the highest densities in the ventromedial parts. The medial occipital cortex was markedly more labeled compared to the rest of the cerebral cortex, whereas binding densities were very low in the cerebellar cortex and in the thalamus. The lower brain stem and spinal cord were not included in the study, while no information was gained on [^11^C]zolmitriptan binding to the raphe nuclei and trigeminal spinal nuclei.

Previous studies have localised 5-HT_1B_ receptors on serotonergic and nonserotonergic neurons, acting as presynaptic auto-and heteroreceptors, respectively, putatively regulating neurotransmitter release [20–22]. 5-HT_1B_ receptor mRNA has been detected in raphe nuclei, striatum, cerebellum, hippocampus, entorhinal and cingulated cortex, subthalamic nucleus, and nucleus accumbens, but not in the substantia nigra or globus pallidus [23–28]. Autoradiographic visualization of 5-HT_1B_ receptors was found in partly different areas, including dense packing in ventral pallidum, globus pallidus, substantia nigra, dorsal subiculum and moderate dense packing in cerebral cortex, molecular layer of the hippocampus, entopeduncular nucleus, superficial gray layer of the superior colliculus, caudate putamen, and deep nuclei of the cerebellum [29–33]. This mismatch between synthesis and localization is explained by hypothesizing that this receptor is synthesized at a different place (cell body) and transported from there to axon terminals, both in serotonergic and nonserotonergic neurons [34].

Similar to 5-HT_1B_ receptors, binding sites attributed to the 5-HT_1D_ receptor are present in globus pallidus, substantia nigra, caudate and putamen, hippocampus, and cerebral cortex., whereas 5-HT_1D_ receptor mRNA is expressed at low levels in the basal ganglia, dorsal raphe nucleus, and locus ceruleus, indicative of the 5-HT_1D_ receptor being located predominantly on axon terminals of both serotonergic and nonserotonergic neurons [35].

New data point to a role of the basal ganglia in migraine. Functional neuroimaging studies have revealed that the substantia nigra (SN) together with the red nucleus (RN) and the occipital cortex (OC) is activated during the attack [36].

 Autoradiographic studies of [^3^H]sumatriptan (5-HT_1B_, 5-HT_1D_, 5-HT_1F_ receptors) and of [^3^H]GR 125743 (5-HT_1B_ and 5-HT_1D_ receptors) in the human brain [16, 37–39] all showed the highest density of binding sites in the visual cortex, substantia nigra, and medial globus pallidus, with the 5-HT_1B_ receptor being the most abundant receptor subtype in the SN and GP [39]. All five regions tested for differential binding of [^11^C]zolmitriptan to 5-HT_1B_ or 5-HT_1D_ receptors (Table 2), showed 5-HT_1B_ binding to be most abundant, while 5-HT_1D_ receptor binding was increased 2-8-fold in the occipital cortex versus the SN and basal ganglia. 

The specific [^11^C]zolmitriptan binding could only to a small degree (<25%) be displaced by the 5-HT_1D_ antagonist BRL15572, indicating that this receptor is not a major contributor to the efficacy of zolmitriptan. This is consistent with the outcome from a clinical trial of the 5-HT_1D_ receptor agonist [40] and the data of Varnäs et al. [16].

WAY-100635 is a highly selective, silent 5-HT_1A_ receptor antagonist which binds with high affinity to the 5-HT_1A_ receptor, at both presynaptic and postsynaptic sites [41]. The anatomical distribution of [^3^H]WAY-100635 binding has been described in detail using large-scale autoradiography on human hemispheric brain cryosections [42]. High densities were found in the hippocampus, superficial layers of the neocortex, and the raphe nuclei. The distribution of [^11^C]WAY-100635 in the present paper was similar to that of [^3^H]WAY-100635, with high-density binding in the neocortex and the raphe nuclei of the cynomolgus monkey brain by PET imaging [43]. Consistently, we report high density binding of [^11^C]WAY-100635 throughout the neocortex, except for the visual cortex of the occipital lobe.

## 5. Conclusions

In summary, in saturation studies, [^11^C]zolmitriptan showed specific (90%) binding to a population of high-affinity binding sites in the Rhesus monkey brain. [^11^C]Zolmitriptan binding showed a regional distribution to defined brain regions with the highest densities in the ventral pallidum, external globus pallidus, substantia nigra, nucleus accumbens, and the visual cortex. In competitive binding studies, a 5-HT_1B_ selective ligand blocked the majority of [^11^C]zolmitriptan binding across all regions, while 25% of total specific binding was attributable to 5-HT_1D_ receptor binding in the visual cortex. [^11^C]Zolmitriptan and [^11^C]WAY-100635 binding sites were differentially distributed with little or no overlap between anatomical areas.

## Figures and Tables

**Table 1:** The estimated *B*_max⁡_ (receptor density) and Kd (affinity of [^11^C]zolmitriptan) in different brain regions of the Rhesus monkey.

Brain region	*B*_max⁡_ (pmol/g wet tissue)		Kd (nM)	
	Mean	SE	Mean	SE
Ventral pallidum	17.9	2.6	2.2	1.1
External globus pallidus	12.2	1.4	1.3	0.6
Substantia nigra	11.4	1.6	1.0	0.6
Nucleus accumbens	9.5	0.6	1.8	0.4
Visual cortex	9.8	1.5	1.1	0.7
Frontal cortex	5.2	0.8	5.1	2.1
Cerebellum	1.6	0.4	4.9	3.4

SE: standard error.

**Table 2:** Displacement of [^11^C]zolmitriptan binding in the nucleus accumbens (NcA), globus pallidus externa (GPE), substantia nigra (SN), visual cortex (VC), and frontal cortex (FC) by selective 5-hydroxytriptamine (HT)_1_ receptor antagonists at 1 *μ*M concentration. The numbers represent the percentage of receptor displacement compared to control sections.

Brain region	5-HT_1A_		5-HT_1B_		5-HT_1D_	
	Mean	SE	Mean	SE	Mean	SE
NcA	0	16.0	44	3.0	3	1.0
GPE	0	11.5	67	1.0	12	7.5
SN	22	13.0	79	0.5	9	1.5
VC	18	11.3	73	2.0	25	12.1
FC	0	3.0	50	9.5	3	9.0

SE: standard error.
